# Diagnostic technologies for neuroblastoma

**DOI:** 10.1039/d4lc00005f

**Published:** 2025-07-14

**Authors:** Leena Khelifa, Yubing Hu, Jennifer Tall, Rasha Khelifa, Amina Ali, Evon Poon, Mohamed Zaki Khelifa, Guowei Yang, Catarina Jones, Rosalia Moreddu, Nan Jiang, Savas Tasoglu, Louis Chesler, Ali K. Yetisen

**Affiliations:** a Department of Chemical Engineering, Imperial College London South Kensington London SW7 2BU UK yubing.hu@imperial.ac.uk; b Division of Clinical Studies, The Institute of Cancer Research London SM2 5NG UK; c Faculty of Medicine, Imperial College London South Kensington SW7 5NH UK; d School of Electronics and Computer Science, University of Southampton Southampton SO17 1BJ UK; e Institute for Life Sciences, University of Southampton Southampton SO17 1BJ UK; f West China School of Basic Medical Sciences & Forensic Medicine, Sichuan University Chengdu 610041 China jiangnansophia@scu.edu.cn; g Research Center for Translational Medicine, Koc University Istanbul 34450 Turkey; h Children's and Young People's Unit, Royal Marsden NHS Foundation Trust Sutton SM2 5PT UK

## Abstract

Neuroblastoma is an aggressive childhood cancer characterised by high relapse rates and heterogenicity. Current medical diagnostic methods involve an array of techniques, from blood tests to tumour biopsies. This process is associated with long-term physical and psychological trauma. Moreover, current technologies do not identify neuroblastoma at an early stage while tumours are easily resectable. In recent decades, many advancements have been made for neuroblastoma diagnosis, including liquid biopsy platforms, radiomics, artificial intelligence (AI) integration and biosensor technologies. These innovations support the trend towards rapid, non-invasive and cost-effective diagnostic methods which can be utilised for accurate risk stratification. Point-of-care (POC) diagnostic devices enable rapid and accurate detection of disease biomarkers and can be performed at the location of the patient. Whilst POC diagnostics has been well-researched within the oncological landscape, few devices have been reported for neuroblastoma, and these remain in the early research phase and as such are limited by lack of clinical validation. Recent research has revealed several potential biomarkers which have great translational potential for POC diagnosis, including proteomic, metabolic and epigenetic markers such as *MYCN* amplification and microRNAs (miRNAs). Using POC devices to detect high-risk biomarkers in biofluids such as blood and urine, offers a non-invasive approach to diagnosis of neuroblastoma, enabling early screening at a population level as well as real-time health monitoring at home. This is critical to mitigating long-term morbidity associated with late diagnosis and unnecessary treatment, in turn improving outcomes for neuroblastoma patients.

## Abstract 2

Neuroblastoma diagnosis typically relies on several invasive and time-consuming processes. POC testing provides rapid results, and often does not require specialised equipment or training, meaning that it can be used by patients at home.

## Graphical abstract

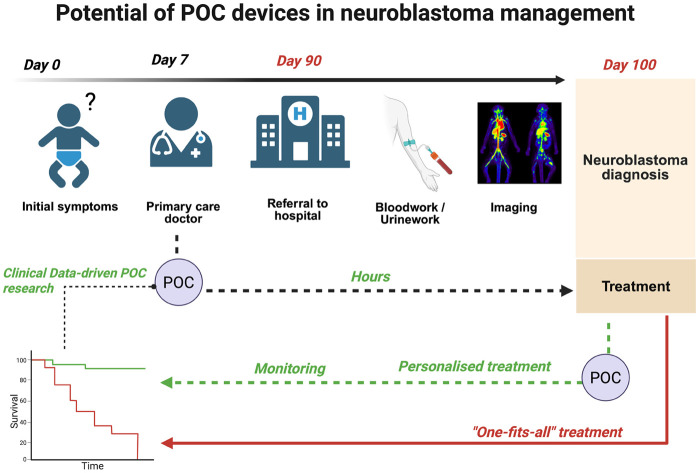

## 1. Introduction

Neuroblastoma, a neural-crest cell-derived malignancy arising from the peripheral sympathetic nervous system,^1^ is the most common extra-cranial solid tumour in children, representing around 5% of all paediatric cancer diagnoses but disproportionally accounting for 15% of childhood paediatric oncology deaths.^2,3^ It is characterised by wide clinical heterogeneity and frequent metastasis.^1^ Neuroblastoma originates from neural crest progenitor cells due to disrupted differentiation processes involving pathways such as WNT, BMPs, and SWI/SNF complexes, resulting in highly heterogeneous tumours.^4–6^ Key genetic drivers include *MYCN* amplification (seen in ∼20% of cases), *ALK* mutations, and *PHOX2B* alterations, all linked to poor prognosis and aggressive disease.^7–12^ Children diagnosed under 1 year of age typically fall into low-risk categories with excellent survival (>95%), whereas high-risk neuroblastoma, often presenting in children over 1 year old, has poor survival and high relapse rates.^13–16^ Predominantly, neuroblastoma is a sporadic disease; however some familial cases have been documented.^17^ Most cases of neuroblastoma are diagnosed following the development of symptoms in children; since tumours arise from tissue of the sympathetic nervous system, typically the adrenal medulla, neuroblastoma is often first suspected due to mass lesions in the abdomen or associated symptoms such as weight loss and breathing difficulties.^18^ Metastatic disease is noted in 60% of neuroblastoma patients, and it is often not until the disease has spread that patients present with symptoms of the disease.^19^ Early diagnosis and accurate risk stratification are essential to the provision of appropriate treatment in patients.^20^ Multimodal treatment approaches include stem cell transplantation, surgery and chemotherapy which induce long-term permanent morbidity.^21^ For neuroblastoma, the most significant prognostic factor is age at diagnosis: earlier diagnosis of disease, whilst tumours are still localised and surgically resectable, contributes to significantly favourable outcomes.^22^ Several attempts at screening young infants for neuroblastoma have been unsuccessful. Elevated urinary catecholamine metabolites homovanillic acid (HVA) and vanillylmandelic acid (VMA) have been indicated for neuroblastoma diagnosis for many decades.^23^ In the early-to-mid 1970s, Japan pioneered a small scale screening programme to measure VMA concentrations in infants at 6 months of age.^24^ Although screening led to increased diagnosis of neuroblastoma, there was no improvement in outcomes for patients. This was because screening detected only favourable characteristics and led to the over-diagnosis of low-risk cases, many of which may have experienced spontaneous disease regression; this contributed to morbidity in patients following unnecessary therapy.^25^ Other similar studies in Canada^26^ and Germany^27^ concluded the same findings. The ability to screen for and diagnose neuroblastoma at an early stage has been a longstanding issue, and the development of highly sensitive, selective, and rapid diagnostic methods is of great importance to improving outcomes for these patients.^28,29^ The success of such a device would be highly dependent on the selection of appropriate biomarkers which are specific to high-risk neuroblastoma and present early in the disease.

Despite the rarity of the disease, the global neuroblastoma market held a value of 1.92 billion pounds in 2016.^30^ The neuroblastoma treatment market was worth over £327m in 2020 and is expected to reach £418m by 2027.^31^ The high cost of multimodal therapy approaches in young cancer patients is a great burden to the National Health Service (NHS) and this highlights the need for initiation of lower intensity and more targeted therapy at an earlier stage.^32^ Further, this will facilitate treatment implementation only where necessary and avoidance of treatment for patients whose disease may spontaneously regress (stage 4S).^33^ Current diagnostic procedures for neuroblastoma involve biochemical profiling, multimodal imaging approaches, and histologic confirmation.^34^ These methods are laborious, invasive, and expensive. Furthermore, none of these methods are appropriate for routine screening of infants for high-risk neuroblastoma before symptoms present.^13^ The introduction of more advanced technologies would allow for more rapid and less invasive diagnosis would revolutionise medical approaches to neuroblastoma. POC tests are often referred to as near patient, bedside or extra laboratory testing.^35^ The main advantages of these techniques over conventional diagnostic methods are reduced length of hospital stay, reduced clinic visits, earlier discharge from hospital and fewer unnecessary hospital admissions.^35^ It also facilitates optimised treatment and mitigates the use of inappropriate drugs, consequently improving longevity and quality of life.^35^ The introduction of POC devices for neuroblastoma would significantly improve diagnosis of paediatric cancers and facilitate the move towards personalised medicine. Recently, there has been a great interest in POC devices for cancer diagnosis,^36^ given that cancer is the most prevalent disease in the world.^37^ It is well-documented that early diagnosis and targeted therapy are essential to providing good outcomes for oncology patients.^38^ For cancer diagnosis, POC devices have shown several limitations;^39^ this means that such devices are not ready to be used commercially, and these issues must be considered when developing new tools for neuroblastoma. Mainly, lateral flow assays (LFAs) and lab-on-a-chip (LoC) technologies have been researched for cancer diagnosis and monitoring.^37^ LFAs are rapid and cost-effective paper-based devices which have the ability to detect very low concentrations of analytes in biofluids.^40^ However, in the cancer field, they are limited to lack of multiplexing, commercialisation, and clinical validation.^41^ Further, LFAs for nucleic acid detection, which is currently an exciting avenue of research, require multiple steps for gene extraction and amplification, thus making them too complex for routine use in clinical settings and at home by patients.^42^ LoC technologies are highly promising, however again they are often limited by the requirement of complex steps, and most attempts to develop such devices for cancer detection will require more optimisation and testing before they can be employed for use in hospitals.^43^ Little to no research has been undertaken for other POC technologies, such as smartphone-based devices, leaving a gap for future research to focus.

Herein, current diagnostic approaches to neuroblastoma and their relative performances and limitations are explored. A comprehensive overview is provided of emerging technologies that are reshaping the diagnostic landscape and paving the way for POC applications. This review also explores POC platforms, and the advantages of these over conventional methods for broader cancer and neuroblastoma diagnosis are considered. Circulating biomarkers in neuroblastoma patients are also investigated and their potential for rapid disease detection. Specifically, the ability to differentially-detect high-risk neuroblastoma using POC devices is of great interest to clinicians and researchers alike, and the future potential and feasibility is explored.

## 2. Neuroblastoma diagnosis

### 2.1 Current clinical methods

**Fig. 1:**
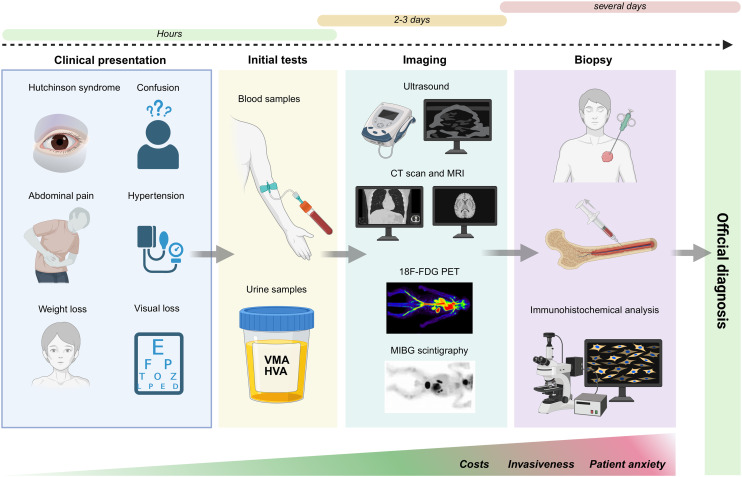
Overview of the current clinical pathway for neuroblastoma diagnosis. Patients often present with non-specific symptoms such as abdominal pain, hypertension, weight loss, or Hutchinson syndrome. Initial diagnostic steps include blood and urine tests (*e.g.*, VMA/HVA levels), followed by imaging techniques such as ultrasound, CT, MRI, 18F-FDG PET, and MIBG scintigraphy. Confirmation requires invasive tissue or bone marrow biopsy and immunohistochemical analysis. The diagnostic timeline spans from hours to several days, with increasing costs, invasiveness, and patient anxiety along the pathway.

Neuroblastoma is often initially identified when a child presents with signs and symptoms of the disease.^19^ Biochemical analyses coupled with multimodal imaging techniques may be undertaken if a patient is suspected of suffering from the disease.^44^ However, ultimately, diagnosis of neuroblastoma is confirmed by either tumour tissue biopsy and histopathologic analysis, or a combination of the detection of neuroblastoma tumour cells in the bone marrow and either elevated urine or serum catecholamines^34^ (Table 1). Here, we have summarised current techniques utilised to diagnose patients with neuroblastoma (Fig. 1). Detailed information will be demonstrated to compare the accuracy of current diagnostic techniques and their relative convenience in diagnosing neuroblastoma. We explore the complexity, cost, advantages and disadvantages, sensitivity, and accessibility of the techniques.

**Table 1:** Summary of conventional diagnostic techniques for neuroblastoma

Diagnostic technique	Cost	Duration	Invasive or harmful?	Is it specific to neuroblastoma?	Can it aid in staging?	Conclusive for diagnosis?
Patient history and physical examination	Low	POC	No	No	No	No
Catecholamine metabolites	Medium	Rapid	Yes	No	No	No
Blood cell count	Medium	Rapid	Yes	No	No	No
Urine cytology	Medium	Rapid	Yes	No	No	No
Liver and kidney function tests	Medium	Rapid	Yes	No	No	No
Ultrasonography (US)	Medium	Rapid	No	No	No	No
Computed tomography (CT)	High	Time-consuming	Yes	No	Yes	No
Magnetic resonance imaging (MRI)	High	Time-consuming	Yes	No	Yes	No
Metaiodobenzylguanidine (MIBG) scintigraphy	High	Time-consuming	Yes	Yes	No	No
Positron emission tomography (PET) scan	High	Time-consuming	Yes	No	No	No
X-ray	High	Time-consuming	Yes	No	No	No
Biopsy	High	Time-consuming	Yes	Yes	No	Yes

#### 2.1.1 Clinical presentation and initial examinations

The clinical presentation of neuroblastoma varies widely depending on age, stage, location of the primary tumour, and presence of metastases. Tumours in the neck may cause Horner syndrome (ptosis, miosis, anhidrosis),^45^ whilst thoracic and pelvic tumours can cause respiratory or bladder dysfunction, repectively.^46,47^ Metastases are present in around 50% of neuroblastoma patients, often affecting the bone marrow, lymph nodes and bones, leading to anaemia, bone pain, and characteristic signs like “raccoon eyes” (Hutchinson syndrome).^48,49^ Systemic symptoms, including abdominal pain, confusion, fever, weight loss, and catecholamine-induced hypertension and flushing, are also common in neuroblastoma patients. Direct compression of optic nerves or the optic chiasm can lead to visual loss or papilledema.^50^ Opsoclonus myoclonus syndrome (OMS) is rare but highly associated with neuroblastoma.^51^ Currently, no environmental factors are known to increase the chances of developing neuroblastoma.^52^ Neuroblastoma is common in infants and extremely rare in those over the age of 10, and familial cases tend to present earlier and involve multiple sites.^53^ Genetic factors such as *BRCA2*, *NRAS* and *TP53* mutations, along with BARD1 and LMO1 polymorphisms, also lead to an increased risk of disease.^2,54^ Some perinatal factors such as maternal anaemia and neonatal complications have been linked to neuroblastoma, although the findings have been inconsistent.^55–66^ When a patient presents with these symptoms, initial assessments include reviewing family history, palpating the abdomen to check for masses, measuring blood pressure, and checking for nodules.^67–70^ Neurological examinations may also be performed such as nerve and pain testing of the arms and legs, since tumours near the spinal cord may cause nerve compression, ultimately affecting sensory or motor functions.^69^

#### 2.1.2 Blood and urine tests

If a patient is suspected of having neuroblastoma, blood and urine tests will be among the first investigations to be undertaken by the clinician given their accessibility and rapidity in producing results (1–2 days). Catecholamine metabolites such as VMA and HVA are elevated in over 90% of neuroblastoma, although they do not differentiate between high and low risk cases. Levels of these metabolites are measured using high pressure liquid chromatography (HPLC) in urine, providing a non-invasive test.^18,71^ Blood analysis may reveal anaemia,^72^ thrombocytopenia,^72^ elevated lymphocyte counts^73^ or other haematological manifestations due to bone marrow infiltration,^74^ although these are not specific to neuroblastoma. Urine cytology is not routinely used but shows promise in the detection of tumour cells and could offer a potential pathway for rapid, non-invasive diagnostics in the future.^75^ Results from liver and kidney function tests help doctors to identify if tumours have spread^76,77^ although, again, they are not conclusive or specific for neuroblastoma diagnosis and obtaining blood samples from children is challenging.

#### 2.1.3 Imaging

Functional imaging plays a key role in the assessment, diagnosis and staging of neuroblastoma. Imaging modalities can be used to identify image defined risk factors (IDRFs) according to the INRG guidelines, for staging and diagnosis.^78^ Imaging of young children often requires general sedation, which must be careful justified due to neurotoxicity risks. US is the first line imaging modality used to investigate patients with a palpable abdominal mass.^67^ It provides a rapid, non-invasive method to analyse tumour location and characteristics and it is able to differentiate neuroblastoma from other abdominal masses.^79^ However, there are several limitations including lack of penetration through dense structures like bone.^80^ Furthermore, US has been found to be less sensitive and accurate for neuroblastoma diagnosis compared to CT and MRI.^81^ CT creates more detailed images of internal body structures and can help identify tumour size, vascular encasement, and metastases.^82–86^ Despite this, major drawbacks include high cost, exposure to ionising radiation and the requirement for general sedation, making CT an invasive procedure for young children.^82^ Furthermore, it has been suggested that the size of adrenal tumours can be underestimated by CT.^87^ MRI has become increasingly preferred as it does not expose patients to ionising radiation. Furthermore, MRI has the advantage of determining marrow infiltration and intra-spinal tumour extension due to overall superior resolution of anatomical detail.^88^ However, adverse effects of gadolinium administered during MRI have been documented.^89^ MIBG scintigraphy is perhaps one of the most important and useful diagnostic tools for neuroblastoma and is routinely used for staging and follow up,^90^ although it is difficult to distinguish between tumour types.^79^ Whilst MRI demonstrates higher sensitivity that MIBG, MIBG offers the great advantage of enhanced specificity for neuroblastoma diagnosis.^21^ More recently, 18F-FDG PET scans has demonstrated high sensitivity (100%) and can detect more lesions that MIBG.^91^ However, this technique was shown to have a relatively low specificity of 50%, and therefore must be used in conjunction with other findings (biochemical or pathological) to ensure accurate diagnosis of neuroblastoma.^91^ Finally, X-rays can be used to show the presence of tumours, however their diagnostic value for neuroblastoma is limited, and the exposure to radiation may be harmful to children.^80^

#### 2.1.4 Biopsies and immunohistochemistry

Biopsies are crucial to confirm a diagnosis of neuroblastoma and for staging of neuroblastoma; CT scans alone have been found to be 82% accurate, whilst CT complemented with biopsy showed 97% accuracy.^92^ Incisional (or open) biopsies provide important information for paediatric tumour cells; however they are invasive and carry surgical risks including infection and gross tumour spillage.^93–95^ Needle (or closed) biopsies such as fine-needle aspiration or core needle biopsy are generally preferred since they are less invasive although they are often guided by CT or US, making them more expensive despite the reduced risks.^96^ The small samples obtained are often insufficient for the completion of all necessary tests, and further do not differentiate neuroblastoma from other tumours.^95^ Bone marrow aspiration is useful, especially for detecting metastatic spread in advanced-stage disease.^97^ Alongside raised urinary or circulating catecholamine levels, positive biopsy results from bone marrow are sufficient to confirm the diagnosis of neuroblastoma. Routinely, bone marrow aspiration and biopsy are performed simultaneously from pelvic bones following local or often general anaesthetic. Following collection of neuroblastoma samples and aspirates, immunohistochemical analysis is a prerequisite for diagnosis confirmation. Therefore, although biopsies provide the most definitive results in terms of diagnosing neuroblastoma, each method presents limitations regarding invasiveness, specificity, and diagnostic completeness.

#### 2.1.5 Downfalls in current techniques

Despite significant advances in neuroblastoma diagnosis, current methods have several limitations which make timely and accurate diagnosis difficult. Blood and urine testing show suboptimal sensitivity, often requiring expensive techniques such as HPLC which are unsuitable for routine POC use.^98^ Imaging modalities include CT, MRI and MIBG scintigraphy. Although these provide critical information for staging and treatment planning, the high costs, radiation exposure, and reliance on sedation make them undesirable for use in paediatric populations.^99^ US is safe and widely available, however cannot offer definitive diagnosis.^99^ Ultimately, positive biopsy findings are required to confirm the diagnosis of neuroblastoma, which are invasive and pose surgical risks, and distinguishing neuroblastoma from other similar tumours can be difficult with many of these techniques.^100^ Altogether, this makes the current diagnostic workflow for neuroblastoma expensive, time-consuming, and invasive, and importantly they are unable to detect neuroblastoma early, before the disease has advanced to a late stage. This highlights the need for cheap, rapid and convenient tools that could be used to screen for children with high-risk forms of neuroblastoma, to avoid significant morbidity associated with late-stage disease as well as for earlier implementation of personalised therapy. The development of POC technologies for neuroblastoma could revolutionise diagnosis and improve outcomes for patients.

### 2.2 Recent advances in neuroblastoma diagnosis

Over recent years, refinements have been made to conventional diagnostic techniques for neuroblastoma, however the methods have remained largely the same and diagnosis relies on imaging and histopathology. There is an urgent need to direct research towards the development of novel technologies that can address the limitations in current methods, for rapid diagnosis and early screening, both of which would play a major role in mitigating the aggressive consequences of this disease. Herein, a summary of recent developments in the diagnosis of neuroblastoma is provided, and how these are paving the way for the creation of rapid and cost-effective tools.

#### 2.2.1 Nuclear imaging

**Fig. 2:**
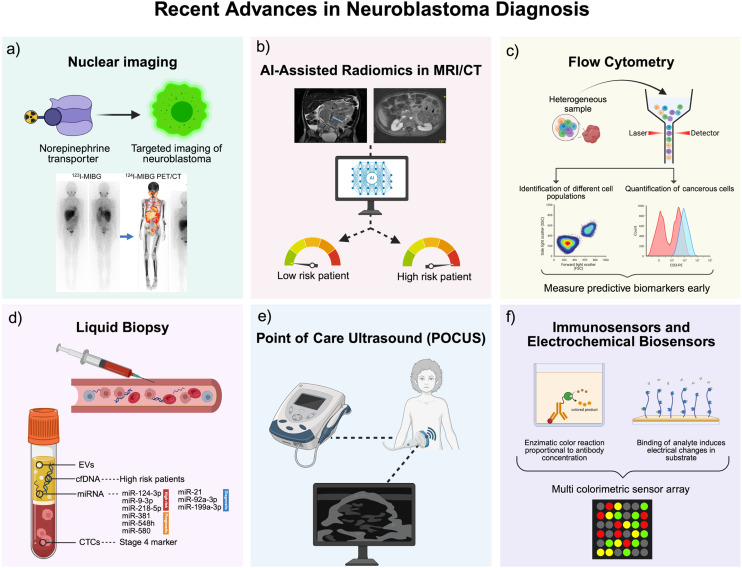
Recent advances in neuroblastoma diagnostics. (a) Nuclear imaging using radiolabelled MIBG enables targeted visualization of neuroblastoma *via* the norepinephrine transporter. (b) AI-assisted radiomics applied to MRI/CT allows risk stratification of patients based on extracted imaging features. (c) Flow cytometry identifies and quantifies cancerous cells within heterogeneous populations to detect early biomarkers. (d) Liquid biopsy captures circulating biomarkers (EVs, cfDNA, miRNAs, CTCs) for non-invasive risk and stage assessment. (e) POCUS offers real-time, bedside imaging to support rapid clinical decision-making. (f) Immunosensors and electrochemical biosensors detect analytes through colorimetric or electrical signals, enabling sensitive multiplexed diagnostics.

Since the early 2000s, nuclear imaging techniques for neuroblastoma have evolved significantly. Imaging has remained foundational in neuroblastoma diagnosis, evolving from primitive techniques to advanced modalities that enhance sensitivity and specificity. Nuclear imaging remains an essential tool in the diagnosis and management of neuroblastoma, particularly for staging at presentation and monitoring treatment response. The standard approach is 123I-MIBG scintigraphy, often combined with SPECT-CT^101^ (Fig. 2a). This technique leverages the norepinephrine transporter (NET) expressed by most neuroblastomas, allowing for targeted imaging. When performed correctly, 123I-MIBG imaging has high diagnostic accuracy, with reported sensitivity and specificity values around 90% for detecting neuroblastoma lesions.^102^ That said, there are important limitations to note. Roughly 10% of neuroblastomas are MIBG non-avid, either due to insufficient NET expression or alterations in uptake mechanisms, which can result in false negatives.^101,103,104^ Also, 123I-MIBG imaging has relatively low spatial resolution, making it less reliable for detecting small lesions, especially in areas with high physiological uptake like the liver or bladder.^105^

To address these gaps, newer PET-based radiotracers are being explored. PET offers clear advantages over SPECT: superior spatial resolution, faster acquisition times (around 15 minutes *versus* 90 for SPECT), better quantification of tracer uptake, and full-body tomographic coverage.^105,106^ Tracers like 18F-MFBG and 123I-MIBG have the same uptake mechanism as 123I-MIBG but benefit from the higher image quality of PET. For instance, 18F-MFBG PET-CT has been shown to detect all lesions seen on 123I-MIBG imaging, plus additional ones, in early human studies.^107^ Similarly, 124I-MIBG PET can improve lesion detection, but has drawbacks such as significantly higher radiation dose—up to 30 times higher than 123I-MIBG in children under 5 years—and more complex logistics due to the tracer's long half-life.^108^ Alternative PET tracers like 18F-FDG and [68Ga]Ga-DOTATATE are especially useful for MIBG-negative cases. 18F-FDG, while not tumour-specific, has shown good sensitivity in such scenarios. In one study, 18F-FDG PET had higher sensitivity (78%) and specificity (92%) than 123I-MIBG (50% and 75%, respectively) for detecting lesions in patients with MIBG-negative tumours or discordant imaging findings.^109^ However, 18F-FDG's use is limited by high physiological uptake in bone marrow and the brain, which can obscure small lesions and lead to false positives, especially after chemotherapy or G-CSF treatment.^110–112^ [68Ga]Ga-DOTATATE PET-CT, which targets somatostatin receptors (primarily SSTR2), has also demonstrated excellent performance. In a study comparing [68Ga]Ga-DOTATATE PET with 123I-MIBG scintigraphy in 42 patients with high-risk neuroblastoma, DOTATATE PET detected more bone and soft tissue lesions in the majority of cases and was positive in all patients, while 123I-MIBG failed to detect disease in two patients.^113^ Moreover, PET imaging with [68Ga]Ga-DOTATATE enables identification of patients eligible for peptide receptor radionuclide therapy (PRRT), offering a theragnostic pathway.^114^

Despite the promise of PET imaging, these newer techniques aren't yet standard in all centres due to limited availability, higher cost, and the need for further validation in larger cohorts. Still, the diagnostic and prognostic potential—especially in MIBG-negative or relapsed cases—is significant. As access to PET tracers improves and more prospective studies are published, these methods are likely to become integrated into standard neuroblastoma management. However, these methods are not likely to support early screening, staging or diagnosis of disease, and are costly and time-consuming, relying on specialised radiotracers and equipment.

#### 2.2.2 AI-assisted Radiomics in MRI/CT

Radiomics and artificial intelligence (AI) have emerged as promising tools for the non-invasive diagnosis of neuroblastoma (Fig. 2b). Radiomics involves extracting large amounts of data from medical images and uncovering patterns that may not be visible to the naked eye, and the implementation of trained AI algorithms allows for this process to be automated.^115^ They have been used to predict *MYCN* amplification, which is a key genetic marker and linked to high-risk disease. In a study by Wu *et al.*^116^ (2020), AI was used to extract seven radiomic features from three-phase CT images and combine these with clinical factors. The nomogram produced achieved an area under the curve (AUC) of 0.95 in the training cohort and 0.91 in the test cohort, where a value of 1 signifies perfect discrimination between healthy and diseased states. This was superior to the clinical model. Another similar study^117^ in 2021 constructed a CT-based radiomics model using logistic regression, support vector machine, and random forest. The study was able to extract more radiomic features (1218) and demonstrated strong performance across all of the models. Building on this, Wang *et al.*^117^ (2021) utilised XGBoost to integrate CT-based radiomic gestures to classify MYCN amplification status. This model achieved an AUC of 0.930 and 0.880 in the training and testing groups, respectively, emphasising the potential of integrating advanced machine learning methods in neuroblastoma imaging. MRI-based radiomics have also been introduced but less widely explored. In 2023, Ghosh *et al.*^118^ reported AUCs of 0.75 and 0.78 using logistic regression and random forest by using MRI-derived features to predict MYCN amplification. The results were promising, but less impressive than the CT-based studies. More recently, Qian *et al.*^119^ used 18F-FDG PET/CT radiomics to predict gene alterations, however the specificity for neuroblastoma was low. Overall, these studies suggest how radiomics and AI models show potential for improving non-invasive high risk neuroblastoma diagnosis. However, it is unlikely that such methods will be available at POC for screening young children, due to the requirement for high quality infrastructure.

#### 2.2.3 Flow cytometry

Flow cytometry (FC) is another recent tool which has great diagnostic value for neuroblastoma that enables quantitative assessment of tumour cells in several biological samples. FC utilises a beam of light (usually laser) to detect and measure the properties of cells, and markers are used to tag specific proteins (Fig. 2c). A study by Furlanetto *et al.* Furlanetto, Spagnol, Alegretti, Farias, Soares, Daudt, Loss, Scroferneker and Michalowski^120^ (2021) found that of 48 samples from 21 patients, FC demonstrated a sensitivity of 100% and specificity of 86% compared to the gold standard of anatomopathological examination (PA) and immunohistochemistry (IHC). In fact, FC was able to identify neuroblastoma cells in samples that PA/IHC could not, highlighting its potential for early detection. A retrospective review at Great Ormand Street Hospital compared FC with cytology and histology for the assessment of bone marrow involvement in neuroblastoma patients. They also found FC to be superior to traditional methods since it was able to identify low-level residual disease.^121^ The identification of minimal residual disease (MRD) by FC was also noted by Cai *et al.*^122^ (2007) through assessment of bone marrow samples. They found that patients who were MRD-negative after treatment had improved outcomes. Similarly, Ye, Wang, Li and Shen^123^ compared FC to cytomorphology (CM) and found that FC also aided in early diagnosis. These findings suggest that FC could be beneficial not only for risk stratification and therapy planning but to support early diagnosis and for prognosis prediction. Moreover, there are attempts to miniaturise and simplify cytometry, such as the development of microfluidic-based FC,^124^ of which a few commercial systems such as the Accelix exist.^125^ This could support early diagnosis and at-home relapse monitoring. However, further research is needed to standardise FC methods and integrate this into routine clinical practice.

#### 2.2.4 Point of care ultrasound (POCUS)

There have been numerous recent cases where POCUS has been utilised as a diagnostic tool in neuroblastoma. In one case, POCUS was able to identify neuroblastoma where numerous other investigations were unable to identify an underlying pathology.^126^ One of the most significant advantages of POCUS is its ability to expedite the evaluation and treatment of patients. The technique is quick, painless, and non-invasive, making it particularly suitable for children (Fig. 2e). Unlike more traditional imaging modalities such as CT scans, POCUS does not involve exposure to ionising radiation, which is a crucial consideration when diagnosing paediatric patients.^127^ A noteworthy characteristic of POCUS in the context of neuroblastoma is its accessibility. In emergency departments, doctors were able to identify abdominal masses in children who presented with non-specific symptoms.^128^ Currently, detailed published studies examining the diagnostic sensitivity and specificity of POCUS for neuroblastoma are limited, making it difficult to form a comprehensive evaluation.^129^ Despite recent promising cases showing the utility of POCUS in the diagnosis of neuroblastoma, it cannot independently diagnose neuroblastoma due to lack of specificity, and is likely to be more useful in combination with other more specific tests. Furthermore, it relies on a trained and experienced person to analyse the results. Nevertheless, it can be used to guide early specialist referral and initiate diagnostic workup.

#### 2.2.5 Liquid biopsy

Since around 2010, liquid biopsy techniques have shown significant promise as non-invasive alternatives to traditional biopsies. By analysing circulating tumour DNA (ctDNA), circulating tumour cells (CTCs), extracellular vesicles (EV) and exosomal miRNAs, these techniques have been investigated for their potential diagnostic and prognostic roles (Fig. 2d). Cell-free DNA (cfDNA) has shown significant promise, especially in high-risk patients. Lodrini *et al.*^130^ reported median cfDNA levels of 94.75 ng mL^−1^ in high-risk neuroblastoma patients at diagnosis, compared to 0.72 ng mL^−1^ in healthy individuals, with levels reaching even higher in patients with bone marrow infiltration (median 113.3 ng mL^−1^), highlighting its association with disease burden and severity. MYCN amplification has been detected in circulating cfDNA with frequencies ranging from 20% to 42% across studies.^131–134^ 5-Hydroxymethylcytosine (5hmC) is a recently recognised epigenetic marker associated with tumorigenesis. It can be profiled in cfDNA isolated from plasma, providing an avenue for non-invasive diagnostics. In neuroblastoma, 5hmC signatures in cfDNA are strongly associated with disease status, tumour burden, and patient outcomes.^135^ A study by Chennakesavalu *et al.*^135^ used chromatin immunoprecipitation and sequencing methods to investigate different neuroblastoma cell lines. They found that low expression of bivalent genes was significantly associated with worse survival in high-risk patients. These findings support cfDNA's role in non-invasive molecular profiling, since liquid biopsy genotyping has been achieved on lateral flow assays^136^ and DNA chips.^137^

Circulating tumour cells (CTCs) have also emerged as strong candidates for diagnosis and prognostication. Kojima *et al.*^138^ isolated CTCs in 8 of 10 newly diagnosed and 3 of 4 relapsed neuroblastoma patients using FACS, with key mutations like MYCN amplification detectable in 12 of 13 patients. Another study by Merugu *et al.*^139^ detected CTCs in 26 out of 42 patients, reporting a mean of 40 cells per mL at diagnosis and 6 cells per mL at relapse. Prognostic relevance is underscored by data showing that 55.6% of stage 4 patients had detectable CTCs compared to none in stage 3, and CTC-positive patients had significantly lower 5 year survival (42%) than CTC-negative patients (90%).^140^ Detection of CTC on several POC platforms is possible and has been achieved for other cancers.^141,142^ It is possible to isolate and detect CTC on point-of-care platforms for cancer diagnosis, such as portable cytometers,^142^ microfluidics^143^ and biosensors.^144^ Thus, research into developing and optimising such devices for neuroblastoma should be a point of focus for the future. Recent advances in molecular diagnostics have highlighted circulating RNAs as promising tools for identifying tumour presence in neuroblastoma.^145^ For instance, elevated levels of tyrosine hydroxylase (TH) mRNA in blood have been associated with advanced disease stages and a higher risk of relapse, supporting its role as a non-invasive marker for neuroblastoma detection.^146^ Moreover, the combined expression of neuroblastoma -related genes—such as PHOX2B, CHGA, DCX, DDC, and TH—in blood and bone marrow has shown strong predictive value for disease progression.^147^ Circulating miRNAs, especially those found within exosomes, have also gained traction as potential biomarkers. Several tumour-specific miRNAs, including miR-124-3p, miR-9-3p, and miR-218-5p, have been identified in the serum of high-risk neuroblastoma patients,^148^ while others such as miR-381, miR-548h, and miR-580 have demonstrated prognostic relevance.^149^ Notably, exosomal miRNAs—like miR-21, miR-92a-3p, and plasma miR-199a-3p—are consistently elevated in neuroblastoma cell lines and patient samples, indicating strong diagnostic potential.^150,151^ These exosomal signatures not only reflect tumour presence but may also shift during therapy, offering a dynamic tool for monitoring treatment response.^133^ Exosomes are isolated from blood using ultracentrifugation or commercial kits, followed by RNA extraction and analysis *via* qPCR or NGS, which are expensive, time-consuming and require trained personnel. Given the increasingly important role of miRNA as biomarkers for cancers, POC platforms for their detection have gained attention in recent years to overcome the limitations of current methods.^152^ However, such devices have not yet been developed for neuroblastoma. Since these biomarkers are present in the blood, and can offer selective diagnosis of high-risk disease, their contribution to next-generation POC diagnostic devices for neuroblastoma could be great.

Despite encouraging results, liquid biopsy in neuroblastoma still faces a number of limitations that need to be addressed before routine clinical implementation. One of the main challenges is the lack of standardisation across methodologies—different studies use various kits, detection platforms, and thresholds, making it difficult to compare results or define clear diagnostic cut-offs. Additionally, many studies do not report sensitivity and specificity figures, relying instead on associations with known tumour features. Biological variability, such as inconsistent shedding of tumour DNA or cells, can lead to false negatives, particularly in early-stage disease or minimal residual disease scenarios. Furthermore, most studies have relatively small sample sizes and are often single centre, which limits generalisability. As the field moves forward, larger multicentre trials and the adoption of standardised protocols will be crucial to translating these findings into clinical practice.

#### 2.2.6 Immunosensors and electrochemical biosensors

The development of immunosensors and electrochemical biosensors is a promising approach for rapid, sensitive and minimally invasive neuroblastoma diagnosis, with the potential for POC applications (Fig. 2f). These devices can quantify targets from small sample volumes. One such application is the development of sensors for catecholamine detection. The measurement of urinary catecholamine metabolites—specifically vanillylmandelic acid (VMA) and homovanillic acid (HVA)—has long been a hallmark in neuroblastoma diagnostics. Studies indicate that these markers possess sensitivities exceeding 90% for the disease, although specificity may be compromised due to their elevations in other conditions^153,154^ alongside other diagnostic indicators. Recent studies have showed increased sensitivity (95%) when all eight metabolites – VMA, HVA, 3-methoxytyramine (3-MT), dopamine, epinephrine, metanephrine, norepinephrine, and NMN – are combined.^155^ In recent decades, some novel methods for measuring urinary catecholamines have been explored. In 2021, an AI-enabled multi colorimetric sensor array was developed.^156^ Colorimetric fingerprints were generated by gold nanorods and used as signal transducers which produced colour when exposed to neuroblastoma-associated metabolites. The colorimetric patterns were analysed through machine learning algorithms and were able to differentiate between neuroblastoma and control samples. This technique also produced quantitative results, with very low limits of detection of 022 μM and 0.29 μM for HVA and VMA, respectively. This proves the potential of AI for improved diagnosis, despite the requirement of validation in larger cohorts. Shetty *et al.*^157^ developed a novel electrochemical sensing system that enabled real-time monitoring of dopamine secretion from neuroblastoma cells. This is valuable for studying tumour biology and for monitoring treatment, and the sensor was highly stable, and the results were reproducible, with a low limit of detection. This is promising for *in vitro* diagnostics, and future validation in blood, where tumour cells are present, could make this viable for POC diagnostic and monitoring. Later in 2024, a POC electrochemical sensor was developed for the detection of HVA, which utilised a nickel-doped zinc oxide nanoparticle-modified carbon paste electrode.^158^ The sensor produced results rapidly and required minimal sample preparation, making it portable. Furthermore, it was low cost, highly sensitive and had a low limit of detection. Despite its promise, further validation in larger cohorts would be required, and the technology would need to integrate into user-friendly POC platforms (handheld, disposable device) in order to be used by patients and clinicians alike. Multiplexing for the detection of several neuroblastoma metabolites could also be a promising improvement in the future.

In 2023, Chen *et al.*^159^ developed an electrochemical immunosensor which targeted the disialoganglioside GD2 which is expressed on neuroblastoma cells. They designed a graphene/gold nanoparticle (AuNP)/GD2 antibody-functionalised electrochemical biosensor capable of detecting GD2-positive cells in bone marrow samples. The sensor demonstrated high sensitivity and could detect cells in the range of 10^2^ to 10^5^ cells per mL, and the results matched 100% with gold-standard immunocytochemical staining techniques. Whilst interesting, the requirement for bone marrow samples means painful and invasive procedures for children and thus is not useful in terms of POC testing or rapid diagnostics. Nonetheless, these advancements prove how new-generation technologies are providing promising results that mitigate some of the disadvantages of conventional techniques. Graphene quantum dots (GQDs) have also been used for *in vivo* imaging of neuroblastoma.^160^ Anti-GD2/GQDs were shown to accumulate in neuroblastoma tumours in mice, which enabled fluorescence imaging of the tumours. The combination of nanotechnology with immunosensing could be an interesting avenue of research for future diagnostics, although unlikely to support POC use.

#### 2.2.7 The move towards POC diagnosis

For neuroblastoma, a transformative shift has been observed over the last two decades from conventional, hospital and laboratory-based imaging and histopathology, towards data-driven, molecular, and minimally invasive methods. This is driven by the demand for screening tools, earlier diagnosis, treatment stratification, treatment efficacy monitoring, and monitoring of patients in remission. Future research must prioritise detection of high-risk disease, where early diagnosis would transform outcomes for this aggressive disease. Convergence of AI-powered analytics, biosensor engineering, and minimally invasive biomarker discovery will drive future diagnostics, enabling faster decision-making, more precise risk stratification, and ultimately improving outcomes for children with neuroblastoma.

## 3. POC technologies

Diagnosing neuroblastoma remains a complex and resource-intensive process. Many patients, often young children, undergo invasive procedures or are exposed to ionising radiation simply to confirm a diagnosis.^161,162^ These methods are not only costly and time-consuming, but also emotionally and physically consuming for both patients and their families.^163,164^ Recent research has focused efforts in creating more rapid and convenient tests. However, even so, current diagnostic approaches often fail to differentiate between low- and high-risk forms of the disease.^165^ This makes them ineffective for early screening and, in some cases, may lead to overtreatment or delayed intervention. The goal has been to decrease therapy for low-risk patients to avoid long-term complications while increasing and targeting therapies for high-risk patients to improve overall survival.^166^ POC technologies have the potential to address these limitations. Designed to deliver diagnostic results quickly and closer to the patient, POC tools can bypass the need for centralised labs or highly specialised equipment.^167^ While they've gained traction in infectious disease and chronic condition monitoring, POC devices for neuroblastoma are not yet available.^168,169^ Still, the idea of a rapid, low-cost, and non-invasive diagnostic tool for neuroblastoma is compelling—and increasingly feasible. Such tools could help reduce diagnostic delays and limit the physical and psychological strain of the current clinical pathway. The COVID-19 pandemic accelerated interest in POC technologies by demonstrating their ability to provide rapid, decentralised testing at scale.^170^ Since then, advances in areas like microfluidics, biosensing, and device miniaturisation have led to new generations of portable diagnostic platforms.^171^ Many of these can now deliver quantitative results and are even compatible with smartphones, making them more user-friendly and accessible in non-clinical settings.^172,173^ With POC devices, a diagnosis can often be made in a single visit, allowing for faster treatment decisions. This is particularly beneficial in regions where access to pathology services is limited, or where time is a critical factor.^174,175^ Distributing POC tools through primary care providers, pharmacies, or directly to patients could expand access and reduce the overall burden on healthcare systems.^176^ Although there are no neuroblastoma-specific POC devices currently available, the technology landscape is diverse and growing. POC platforms now include a range of systems—from lateral flow assays (LFAs)^177,178^ and lab-on-a-chip (LoC) devices^179,180^ to electrochemical^181,182^ and optical biosensors,^183,184^ many of which are increasingly being integrated with smartphones. Each of these offers unique advantages, and their continued development could play a significant role in transforming how neuroblastoma—and other many other cancers—are diagnosed and managed in the future.

### 3.1 Types of POC devices

#### 3.1.1 Lateral flow assays

**Fig. 3:**
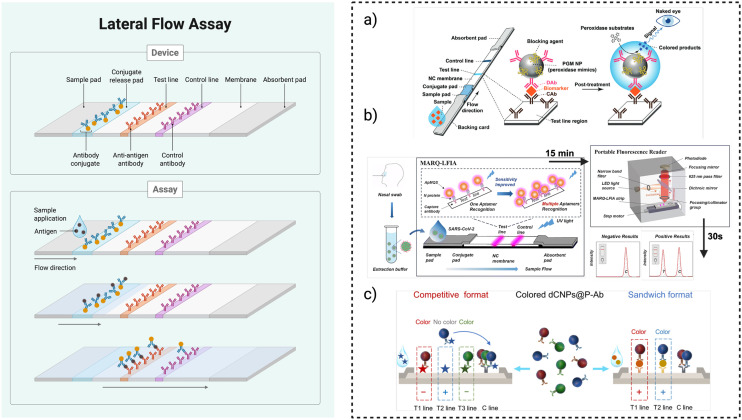
Schematic of a general LFA device and examples of application. (a) Schematic of a colorimetric LFA enhanced by platinum-group metal (PGM) nanoparticles with peroxidase-like activity. The PGM NPs are functionalised with antibodies to form detection probes that bind target analytes, enabling sensitive signal amplification at the test line *via* catalytic colour development after a post-treatment step. Reproduced from ref. 196 with permission from Demetrios A. Spandidos Ed. & Pub., copyright 2017. (b) MARQ-LFIA platform for SARS-CoV-2 detection. Nasal swab samples are applied to a lateral flow strip functionalised with a multiple aptamer recognition-based quantum dot fluorescent probe (ApMQS). Multiple aptamers target distinct epitopes on the viral N protein, enhancing sensitivity. A portable fluorescence reader captures test and control line intensities within 30 seconds, enabling rapid and highly sensitive point-of-care diagnosis. Reproduced from ref. 197 with permission from Royal Society of Chemistry, copyright 2025. (c) Coloured dCNPs@P-Ab used in competitive and sandwich-format multiplex lateral flow assays (mLFAs). In the competitive format (left), increasing analyte concentration reduces the test line signal, while in the sandwich format (right), signal intensity correlates with target concentration. Distinct colors enable visual differentiation of multiple targets at separate test lines. Reproduced from ref. 198 with permission from ACS Publications, copyright 2025.

LFAs are widely used in hospitals, clinics, and laboratories due to their ease of use, affordability, and ability to deliver results within 5–30 minutes. These tests rely on capillary action, which drives the liquid sample across a series of overlapping membranes mounted on a backing card for structural support^185^ (Fig. 3). First, the sample is applied to a sample pad that contains buffer salts and surfactants to optimize flow and stabilize the sample. It then moves into a conjugate pad, where labelled antibodies—typically conjugated to coloured or fluorescent particles like colloidal gold—bind to the target analyte if present. This mixture flows into the detection zone, usually composed of a nitrocellulose membrane, where specific capture antibodies or antigens are immobilized in defined lines.^186^ These capture agents trap the analyte-labelled antibody complexes, forming visible test and control lines. Finally, the fluid is drawn into an absorbent pad that maintains the flow and ensures the test completes properly. Results can be read by eye or with a reader. Attention to all components—including the backing card, adhesive layers, and cover tape—is crucial to ensure consistent, high-quality performance.

To improve the sensitivity and performance of LFAs, a range of new materials and technologies have been developed that enhance how targets are captured and how signals are read^186^ (Fig. 3a–c). Quantum dots, for example, are nano-scale fluorescent particles that can be chemically linked to several biological probes.^187,188^ When they bind to a specific target analyte and reach the test line, they emit a strong, stable light signal under LED or UV light, making it possible to detect even very low concentrations of the target. Magnetic nanoparticles are used earlier in the process: they are coated with molecules that bind to the target analyte and then concentrated using a magnet before the sample is applied to the strip.^189–191^ This “pre-enrichment” step increases the number of target molecules reaching the test line, boosting sensitivity in complex samples like blood or saliva. In addition, label-free sample enrichment methods such as dialysis with polyethylene glycol (PEG) remove interfering molecules from the sample, helping to reduce background noise and concentrate the analyte. Similarly, ion concentration polarization (ICP) uses a small electric current to trap and concentrate charged target molecules at the start of the strip before they migrate, increasing the signal strength.^192,193^ Lastly, modifying the shape and structure of nanomaterials—such as using star-shaped gold nanoparticles instead of spherical ones—has been shown to improve binding at the test line and produce stronger signals.^194,195^

#### 3.1.2 Lab-on-a-chip

**Fig. 4:**
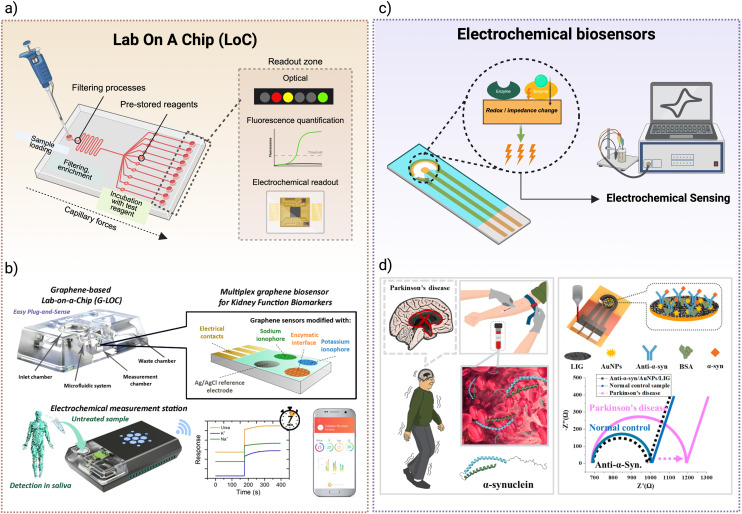
Point-of-care diagnostic devices. (a) Schematic of a lab-on-a-chip (LoC) device utilizing capillary-driven flow through microfluidic channels. Detection zones support both optical and electrochemical readouts for versatile biomarker quantification. (b) Graphene-based lab-on-a-chip (G-LOC) platform for multiplexed, non-invasive detection of kidney function biomarkers (*e.g.*, Na^+^, K^+^, urea) in saliva. Graphene sensors are functionalised with ionophores and enzymatic interfaces, enabling real-time monitoring *via* a mobile electrochemical station. Reproduced from ref. 212 with permission from ACS Publications, copyright 2024. (c) Electrochemical biosensors use enzymatic reactions to induce redox or impedance changes upon target binding. These electrical changes are quantified *via* portable potentiostats or impedance analyzers, offering rapid and sensitive detection at the point of care. (d) Electrochemical immunosensor for the detection of α-synuclein in Parkinson's disease. The platform integrates a laser-induced graphene electrode modified with gold nanoparticles and antibodies, enabling label-free impedance-based detection with high sensitivity. Reproduced from ref. 213 with permission from Elsevier, copyright 2025.

While LFAs are widely used for simple, low-cost diagnostics, LoC devices offer improved analytical precision, flexibility, and automation, making them a promising alternative for more complex point-of-care testing.^179,199^ LoCs miniaturise laboratory functions onto a single chip and allow small fluid samples—such as blood or saliva—to move through microchannels that guide them through various assay steps (Fig. 4a). Depending on the design, samples are either injected using a syringe (active systems) or drawn in automatically by capillary forces, surface tension, pressure, gravity-driven flow or hydrostatic flow (passive systems).^200^ What makes LoCs particularly powerful is their ability to control fluid timing and routing without electronics. This is achieved through design features such as channel geometry, where longer or serpentine channels delay flow; capillary stop valves and burst valves, which hold fluids in place until enough pressure builds; and surface chemistry, where hydrophilic or hydrophobic coatings determine how and where fluids move.^201,202^ In addition, reagents can be pre-stored on the chip, activating only when contacted by the sample, allowing for complex workflows such as mixing, incubation, washing, and detection to occur in sequence. Compared to LFAs, which generally perform one-step, qualitative assays, LoCs enable automated, multi-step, quantitative diagnostics—including ELISAs or nucleic acid amplification—from a single drop of sample. Recent innovations have also improved portability and affordability, including freeze-dried reagents for better shelf-life, smartphone-based readouts, and low-cost fabrication methods like 3D printing^203,204^ (Fig. 4b). Together, these advances make LoCs a powerful and versatile platform for POC diagnostics.

#### 3.1.3 Electrochemical biosensors

In contrast to LFAs and lab-on-a-chip platforms, which rely primarily on optical signals or capillary flow, electrochemical sensors detect analytes by converting biochemical interactions into electrical signals, offering a highly sensitive and inherently quantitative alternative for POC diagnostics.^182,205^ These systems typically consist of a recognition element—such as an enzyme, antibody, or aptamer—immobilized on the surface of an electrode (Fig. 4c). When the target analyte binds to this surface, it induces an electrochemical reaction (*e.g.*, redox or impedance change), which is measured as current or voltage. Electrochemical sensors can be easily miniaturised, require minimal sample preparation, and are well-suited to real-time monitoring, even in wearable formats. Recent technological advances have focused on improving electrode performance using nanomaterials like carbon nanotubes, gold nanoparticles, graphene, and Prussian blue, which enhance conductivity and increase the surface area for biomolecule attachment^206–208^ (Fig. 4d). These modifications allow for greater sensitivity and faster response times, even at a very low analyte concentration. Other innovations include enzyme stabilization methods using nanostructured films or hydrogels, and signal amplification strategies using redox cycling in immunosensors.^209,210^ Additionally, electrochemical sensors are increasingly being developed for non-invasive sampling, using fluids like saliva, sweat, or tears, and incorporated into wearable devices such as flexible skin patches, tattoo sensors, or contact lenses.^211^

#### 3.1.4 Optical biosensors

**Fig. 5:**
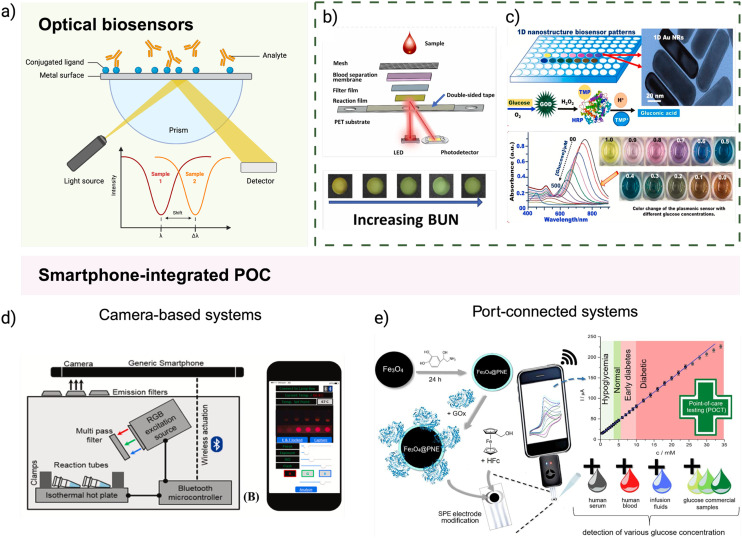
Point-of-care diagnostic devices. (a) General schematic of an optical biosensor: a light source excites the sample at a functionalised sensing surface, where the interaction with specific analytes induces measurable changes in optical properties (*e.g.*, absorbance, fluorescence, or refractive index). These changes are then detected by a photodetector for signal quantification. (b) Paper-based biosensor for blood urea nitrogen (BUN) detection using an integrated optical film and photodiode-based detection unit. Color intensity from enzymatic reactions is quantified digitally, enabling rapid and low-cost clinical assessment. Reproduced from ref. 229 with permission from Elsevier, copyright 2023. (c) Plasmonic glucose biosensor featuring self-assembled anisotropic gold nanoparticles on a paper substrate. Glucose-induced etching alters nanoparticle shape and color, enabling semi-quantitative visual analysis with high stability in biological fluids. Reproduced from ref. 230 with permission from Elsevier, copyright 2021. (d) Smartphone-based POC diagnostic platform using a 3D-printed chamber with integrated RGB LEDs and a smartphone camera. Fluorescent lateral flow strips can be inserted for real-time image-based quantification of nucleic acid amplification signals. Reproduced from ref. 231 with permission from Elsevier, copyright 2019. (e) Electrochemical glucose biosensor with a smartphone-connected interface. A screen-printed carbon electrode is modified with Fe_3_O_4_–poly(thiophene) nanocomposites to enable sensitive and stable glucose detection in saliva, urine, and blood. Reproduced from ref. 232 with permission from Elsevier, copyright 2022.

Optical sensors generate signals based on light absorption, emission, reflection, or interference when a target analyte binds to a biological recognition element^214^ (Fig. 5a). These sensors are often label-free, real-time, and highly sensitive, making them well-suited for rapid diagnostics. Common formats include fluorescence, colorimetric, surface plasmon resonance (SPR), and interferometric biosensors, each offering unique advantages depending on the application.^184,215,216^ New materials such as gold and silver nanoparticles, quantum dots, photonic crystals, graphene, metal–organic frameworks (MOFs), and biocompatible polymers have been employed to improve signal strength, specificity, and multiplexing^217–219^ (Fig. 5b and c). For example, plasmonic nanomaterials like gold nanoparticles enhance the sensitivity of SPR-based sensors by amplifying the change in refractive index upon analyte binding, while quantum dots provide bright fluorescence for detecting multiple biomarkers simultaneously. Colorimetric sensors, which produce visible colour changes, are particularly useful for low-cost, naked-eye readouts and are often combined with smartphone cameras for semi-quantitative analysis. Additionally, flexible and wearable optical sensors, such as tattoo-based sensors or smart patches, enable continuous, non-invasive monitoring of glucose, cortisol, and other health indicators in sweat or interstitial fluid.^220^ Emerging platforms like lab-on-chip optical sensors and implantable sensors further extend their capabilities, integrating complex detection mechanisms with miniaturised fluidics for real-time *in vivo* analysis.^220^

#### 3.1.5 Smartphone-integrated POC

Smartphone-integrated POC diagnostics have emerged as a powerful extension to conventional POC technologies, combining sensor-based detection with real-time connectivity and user-friendly interfaces.^221^ Unlike LFAs, LoC devices, or stand-alone biosensors, smartphone-based platforms offer an all-in-one solution for sample acquisition, detection, analysis, and data sharing (Fig. 5d and e). These systems operate by either using the smartphone's built-in features—such as cameras, microphones, and flash LEDs—or through external attachments like optical cradles, electrochemical modules, or microfluidic adapters.^222,223^ They can support both *in vivo* sensing (*e.g.*, monitoring heart rate or skin images) and *in vitro* diagnostics (*e.g.*, detecting biomarkers in saliva, blood, or urine) using various detection principles including colorimetric, fluorescence, electrochemical, and spectrometric assays.^223–225^ Recent advancements have focused on improving detection sensitivity, portability, and automation by integrating nanomaterials, microfluidics, and miniaturised optics into compact adapters that align with smartphone cameras. These platforms also leverage software innovations, such as machine learning algorithms and cloud-based databases, to enhance result interpretation, pattern recognition, and remote clinical feedback.^226,227^ Importantly, smartphones allow direct data sharing *via* Wi-Fi, Bluetooth, or mobile networks, enabling real-time communication with healthcare professionals, centralized labs, or epidemiological tracking systems. In resource-limited settings, smartphone-based POC tools provide affordable, decentralispered diagnostics without requiring full laboratory infrastructure.^228^ Applications span across infectious diseases (*e.g.*, COVID-19, malaria), chronic conditions (*e.g.*, diabetes, cardiovascular diseases), reproductive health, and even fertility screening. Their low power needs, high accessibility, and compatibility with wearable biosensors make smartphones a key enabler of mobile health systems, supporting personalised medicine and continuous patient monitoring outside of clinical environments.

### 3.2 POC technologies in cancer research

Diagnosing cancer is rarely straightforward, particularly in its early stages. Most cancers don't cause clear symptoms until the disease is already in a highly advanced state. When symptoms do appear, they're often vague or easily mistaken for more common conditions, which delays proper diagnosis.^233^ Once a diagnosis is suspected, patients typically go through a series of tests. These are effective but depend on skilled personnel, complex equipment, and can take days or even weeks.^234^ Biological variability—tumour heterogeneity—adds another layer of complexity, making it difficult to rely on a single biomarker for diagnosis or prognosis.^235^

**Fig. 6:**
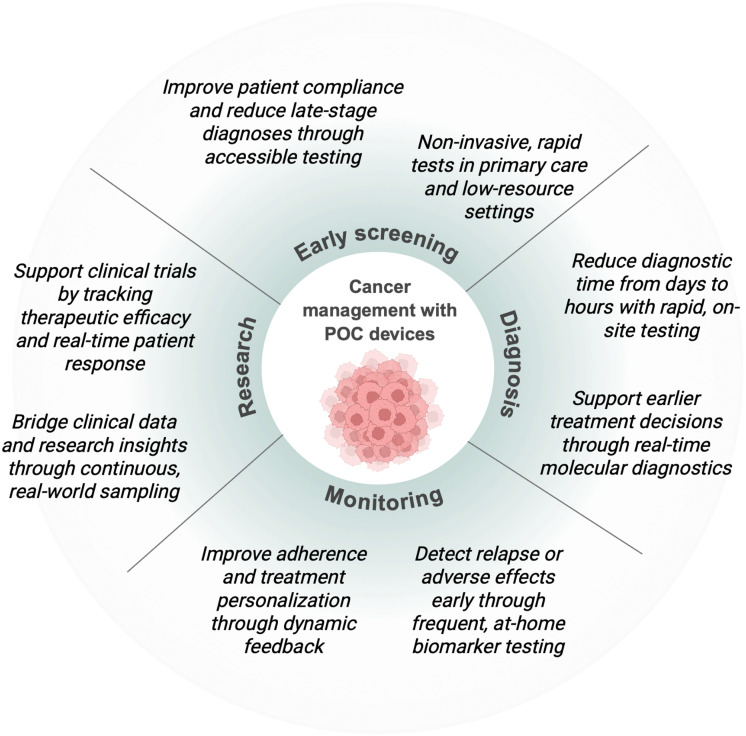
Schematic representation of how point-of-care (POC) devices can enhance cancer management across the care continuum. POC technologies support early screening through accessible, non-invasive tests; enable faster, real-time diagnosis; facilitate treatment monitoring *via* at-home biomarker tracking; and contribute to research by generating high-frequency, real-world clinical data. Together, these applications promote timely interventions, personalised care, and improved outcomes in oncology.

POC technologies offer a promising solution for these challenges (Fig. 6). They often work with easily accessible samples like blood, saliva, or urine, and can be used outside of a hospital setting.^35^ For patients in remote or underserved regions, this kind of access could be transformative, helping detect disease earlier and supporting more regular monitoring.^176^ Lateral flow assays, widely recognized for their affordability, rapidity, and ease of use, have been developed for the detection of protein biomarkers such as prostate-specific antigen (PSA), cancer antigen 125 (CA125), and carcinoembryonic antigen (CEA).^236^ Paper-based microfluidic devices, or μPADs, are another exciting development. These disposable, low-cost platforms can detect cancer cells or DNA using colour change or fluorescence.^237^ In one case, a μPAD combined with a smartphone-based reader successfully identified ROR1-positive cells in blood—bypassing the need for flow cytometry.^238^ More advanced technologies are also emerging. CRISPR-based diagnostics, aptamer-modified sensors, and hybrid electrochemical–optical devices are all being explored for cancer detection.^239^ Genetic and epigenetic markers like methylated DNA and miRNAs are gaining traction too, given their potential to offer insight into tumour subtype or treatment resistance. A recent platform was able to reach a limit of detection as low as 0.16 pM miRNA.^240^ Other cancer LFAs for detection of cancer biomarkers DNA methylation,^74,75^ single nucleotide poylmorphisms,^241^ and circulating tumour DNA (ctDNA) have also been developed; all of which are implicated as biomarkers for several cancers. Kalligosfyri *et al.*^137,242,243^ developed a rapid gold nanoparticle-based LFA for visual detection of KRAS gene mutations in ctDNA, relevant to colorectal cancer, using streptavidin-biotin affinity and four primer extensions at the test zone.

Commercial examples of cancer POC tests are still rare, but a few exist. A one-step PSA test has been developed, and Abbott's NMP22 BladderCheck test can detect a urinary bladder cancer marker in under 30 minutes using just a few drops of urine.^244,245^ However, both have faced criticism due to poor sensitivity and specificity, highlighting the need for further optimization, namely through adding washing steps to reduce background noise, Additionally, relying on a single biomarker is often not sufficient for reliable cancer detection.^235^ As a result, interest is growing in multiplexed lateral flow assays that can detect several targets simultaneously.^246^ When paired with smartphone-based readers, these multiplexed devices could offer more accurate and accessible diagnostics. LoC has been used to measure miRNAs,^247,248^ detect circulating tumour cells, and analyse markers such as CEA and EGFR in lung cancer.^249^ Compared to traditional LFAs, LoCs can handle more complex assays and often don't require amplification steps—making them ideal for molecular diagnostics. One recent study demonstrated a microfluidic chip that could detect tumour-associated miRNAs in serum without amplification, achieving high specificity and a detection limit below 40 pmol L^−1^.^247^ Wearable cancer diagnostics, however, are still in early stages, with some interesting proof of concepts developed, but with few *in vivo* and clinical validation. Calderón-Santiago *et al.* used sweat to screen for lung cancer,^250^ while the GILUPI CellCollector® captured circulating tumour cells (CTCs) directly from the blood.^251^ Lai *et al.*^252^ enhanced CTC capture using a graphene oxide nano-net, and Park *et al.* developed a microfluidic device to isolate CTCs based on mechanical properties, while Pekin *et al.* detected ctDNA mutations with a microfluidic digital PCR platform.^253^ Beyond initial diagnosis, POC devices hold significant potential for monitoring treatment response, predicting therapeutic efficacy, and identifying early signs of relapse.^254^ For instance, phosphoCDK levels could be tracked during trials involving CDK inhibitors, giving real-time insight into drug activity.^255^ POC devices also lend themselves to time-point sampling, allowing clinicians to track changes in biomarker levels throughout treatment. In the future, they could help predict aggressive disease, monitor resistance patterns, or even anticipate metastasis.

In the context of neuroblastoma, POC detection for the detection of VMA and HVA in urine has been achieved through the use of nickel-doped zinc oxide nanomaterials.^158^ Other POC immunosensors have been developed that detect GD2 (ref. 159) and dopamine release.^157^ As POC platforms continue to evolve and integrate with AI, 3D printing, and mobile technology, they are steadily moving toward a new standard: diagnostics that are not only fast and portable, but also personalised, predictive, and deeply embedded in everyday care. However, while advances in engineering and device miniaturisation are critical, the success of POC technologies ultimately depends on our understanding of the disease itself. In cancer, this means uncovering the molecular events that drive tumour development, progression, and resistance to treatment.^256^ Without reliable biomarkers—whether genetic mutations, epigenetic changes, protein expression patterns, or metabolic signatures—POC tools lack meaningful targets to measure.^257^ As such, progress in cancer biology, including the identification of early indicators of malignancy or predictors of therapeutic response, is essential for informing the design of clinically useful POC diagnostics. The more precisely we understand tumour heterogeneity, microenvironmental cues, and disease evolution, the more targeted and effective these tools can become.

### 3.3 Biomarkers for POC neuroblastoma diagnosis

POC offer the promise of rapid testing and immediate clinical decision support directly at the site of patient care. The potential for POC systems to utilise nanotechnology for biomarker detection is gaining traction within the oncological landscape, including neuroblastoma diagnosis.^258^ These innovations facilitate timely and accurate diagnoses, which are critical given the aggressive nature of neuroblastoma and the time-sensitive nature of paediatric oncology interventions. Only a few POC devices have been developed for neuroblastoma diagnosis, and these aim to detect catecholamine metabolites. These are still in the early stages of development and, whilst not invasive, do not differentiate between high risk and low risk disease – something which will be essential to the development of an effective screening and relapse monitoring tool. In this section, possible biomarkers for neuroblastoma are explored, and the potential to apply these to different POC platforms, and the concentrations at which these should be measured, is investigated (Table 2).

**Table 2:** Summary of key neuroblastoma biomarkers, including sample type, detection methods, and diagnostic relevance

Biomarker type	Example biomarker	Sample type	Detection method	Stage (early/late)	Key features
Genetic biomarkers	MYCN amplification	Blood	FISH, PCR, cfDNA analysis	Early	Associated with high-risk NB, poor prognosis
	ALK mutation/amplification	Blood	FISH, PCR, cfDNA analysis	Early	Promotes MYCN activity; poor survival
	NSE (neuron-specific enolase)	Spinal fluid, serum or urine	Blood assay	Late	Decreased survival indicator
	Circulating tumour DNA (ctDNA)	Blood	Next-generation sequencing (NGS), dPCR	Early and late	Detection of tumour-specific mutations; useful for monitoring and relapse prediction
	cfDNA	Blood	qPCR, ddPCR-method, whole genome sequencing	Early	Common alterations detected non-invasively; correlated with NB stage
	Ferritin	Blood	Blood assay	Early	Associated with worse prognosis
	LDH	Blood	Blood assay	Late	Increased metastatic burden
	ATRX	Tissue biopsy	IHC, genome sequencing, FISH	Late	Loss of ATRX associated with poor prognosis
	TERT	Tissue biopsy, blood	FISH, RT-qPCR, NGS	Late	TERT mutations linked with aggressive tumour features
RNA biomarkers	TH mRNA	Blood	RT-qPCR	Early	High in advanced stages, relapse predictor
	PHOX2B mRNA			Early	Strong prognostic marker
Circulating MiRNA	miR-124-3p	Blood	RT-qPCR	Early	Regulates PI3K/AKT, TGF-β, and p53 signalling, impacting cell proliferation, apoptosis, and metastasis
	miR-9-3p			Early	Prognostic for high-risk NB
	miR-21			Late	Increased to enable escape from immune detection
	miR-16-5p			Early	Regulates apoptosis
	miR-21-5p			Late	Similar to miR-21, cancer-related, late progression
	miR-125b-5p			Early	Promote tumour cell proliferation and inhibit p53-dependent apoptosis
	miR-199a-3p			Early	Facilitate proliferation and migration
Circulating cells	CTCs expressing GD2, CD90, CD45, and CD235a	Blood	Blood assays	Late	Associated with metastasis, relapse
Metabolic biomarkers	VMA	Urine	HPLC, LC-MS/MS	Early	Classical NB markers; good sensitivity
	HVA			Early	
	3-Methoxytyramine (3-MTS), NMN			Early	Additional urinary markers
	ODC1	Blood, saliva or urine	Western blot, ELISA, IHC	Late	Tumour progression, aggressiveness, poor prognosis
	HLA-G	Blood	ELISA	Late	Increased to enable escape from immune detection
	Polyamines	Blood, urine	HPLC, fluorescence	Early	Elevated urine levels can suggest cancer
	Serum cholesterol	Blood	Lipid profile, enzyme colorimetric method	Early	Elevated levels associated with poor prognosis
	NLR	Blood	Full blood count	Early	Elevated NLR suggests inflammation, associated with poor prognosis
	HALP	Blood	Blood tests	Early	Elevated HALP score associated with better prognosis
Exosomal proteins	NCAM, NCL, MYH9, FN1, LTBP1	Blood	LC-MS/MS, ELISA, Western blot	Early and late	High risk patients, role in cell migration, and metastasis
Proteomic markers	Vitronectin	Blood	ELISA, western blot	Early	Elevated levels associated with poor prognosis
	Butyrycholinesterase	Blood	Fluorescent activity assay	Early	Decreased levels associated with poor prognosis
	Neuropeptide Y	Sweat, blood	Electrochemical detection, GMR biosensors	Early	Elevated levels linked to stress
	GD2	Blood	Immunoassay	Early	Can distinguish between high and low risk disease
	Trk	Tissue	IHC, FISH	Early	Trk A associated with good prognosis; Trk B associated with poor prognosis

#### 3.3.1 Genetic and genomic biomarkers

Circulating tumour DNA can be non-invasively assessed in blood or serum, and their implications in diagnosis and prognostication of cancer has been demonstrated.^259^ In neuroblastoma patients, the presence of cell-free DNA (cfDNA) and ctDNA have been documented. Studies have reported that cfDNA is correlated to tumour burden, treatment response and incidence of relapse.^260–262^ Specifically, copy number alterations,^259^ segmental chromosome aberrations,^263^ and mutations or amplifications^132^ in cfDNA can all be used to predict prognosis in neuroblastoma patients and provide a potential avenue for future treatment stratification. So, circulating DNA could be the target for future diagnostic devices to differentiate high risk patients, which will enable personalised treatment of patients, thus working to reduce morbidity related to ineffective therapy. Recently, George *et al.*^264^ have demonstrated a panel-based approach to identify genetic alterations from plasma samples through ctDNA sequencing. This is a promising step towards development of rapid devices for risk and treatment stratification through biomarker monitoring. Later, Stankunaite *et al.*^265^ described the successful novel development of a capture panel to detect genetic variants in cfDNA in children with solid tumours. Detection of mRNA and microRNAs is another recent area of interest to researchers. Studies have suggested that the presence of mRNA of neuroblastoma-associated genes can be linked to tumour stage and clinical outcome.^146,266–269^ Implementation of genetic biomarkers in POC platforms is difficult due to the low abundance of ctDNA and miRNA and the requirement for multiplexing due to genetic heterogeneity. However, advances in microfluidics, smartphone readouts and CRISPR-based detection means these challenges are being actively addressed. Thus, such biomarkers are of great interest.

##### 3.3.1.1 *MYCN* amplification

*MYCN* amplification is the most significant poor prognostic marker in neuroblastoma patients.^270^*MYCN* amplification is present in around 20–30% of neuroblastoma patients, and survival in these patients is less than 50%.^18,271^ Low risk patients lacking *MYCN* amplification can never progress to high-risk disease.^272,273^ In high-risk patients with upregulation of *MYCN*, amplification is always present at diagnosis, thus, amplification of *MYCN* is an early factor which is perhaps driving development of aggressive phenotypes in young patients. Traditionally, fluorescent *in situ* hybridisation (FISH) and southern blot analysis are used to measure *MYCN* copy numbers in tissue samples.^11^ This presents with many technical difficulties, such as interference of cells and other tissue components leading to false negative results, as well as lack of reliability of results due to small DNA quantities. More recently, real-time PCR has been used to quantify *MYCN* copy number in ctDNA.^132^ Detection of *MYCN* amplification is thus difficult, time consuming and requires biopsy samples, however PCR-based techniques are adaptable to portable microfluidic POC devices. Currently, its measurement is only indicated once patients have been diagnosed with neuroblastoma, for the purpose of risk and treatment stratification. Early detection of *MYCN* amplification for screening could be achieved through the detection of MYCN protein in biofluids such as blood.^274^ However, there are currently no confirmed reference ranges for the concentration of the protein in healthy patients. This would be essential to know before a device to detect it could be developed. Furthermore, it is not yet known whether levels of this protein are elevated in neuroblastoma patients, and studies would be required to determine this, although it can be hypothesised that patients with elevated expression of *MYCN* would have higher levels of the protein within circulating tumour cells. When these cells lyse, they may release their contents into the blood stream thus leading to elevated concentrations of the protein in blood. Another issue would be that MYCN protein has a short half-life of around 30 minutes,^274^ and this may present difficulties in determining accurate reference ranges for the protein. One study in 2016 by Smith *et al.*^275^ described the optimisation of an ELISA test for MYCN protein measurement in cell lysates derived from patient bone marrow samples. Devices such as ELISA tests are commercially available for this; however, their use is limited to research purposes and have not been employed for routine clinical use.

##### 3.3.1.2 *ALK* mutations

Anaplastic lymphoma kinase (ALK) codes for a receptor tyrosine kinase and mutations are present in around 14% of high risk forms of neuroblastoma.^276^ Along with PHOX2B, ALK is associated with some forms of familial neuroblastoma. ALK is important for neural development but postnatally, it has little to no expression in healthy tissues. In mouse and zebrafish models, ALK mutations have been correlated with *MYCN* amplification. ALK status is mainly tested for in lung cancer, using techniques such as FISH or other immunohistochemical methods, which are not suitable for POC testing.^277^ Combaret *et al.*^278^ used a droplet digital PCR system to test ALK status in neuroblastoma patients using peripheral blood. This showed promising results with sensitivity and specificity both around 90–100%. Despite this, the process is not suitable for POC testing but is a good alternative to test for ALK status, if there is no tumour tissue available. Developing low cost POC platforms for ALK mutation screening could facilitate early identification of disease.

##### 3.3.1.3 5hmC

5hmC profiling in cfDNA is a recently discovered biomarker for neuroblastoma which could distinguish high risk disease with high sensitivity and specificity. Currently, this technology requires sequencing-based analysis, however there is potential to miniaturise this, for example *via* nanopore sequencing or electrochemical biosensors, thus eventually enabling POC-based risk stratification.

##### 3.3.1.4 *ATRX* mutations and *TERT* rearrangements

Like *MYCN*, mutations in the alpha-thalassemia/mental retardation, X-linked (ATRX) and telomerase reverse transcriptase (TERT) genes are associated with high-risk forms of neuroblastoma.^279^ The majority of these genetic mutations lead to telomere lengthening. ATRX and TERT mutations are more commonly found in patients older than the age of 18 months, with TERT being associated with stage 4 disease and ATRX being associated with a refractory clinical subtype – all of which are unfavourable signs of neuroblastoma. No POC testing of these genes are evident in current literature. The current method for detecting *ATRX* status is through biopsy or surgery, which are invasive, and so currently it is not feasible to transform this into POC.

##### 3.3.1.5 Novel gene biomarkers

Other genes, such as H2AFX, DDX39A, BCL11A and S100A9 have been shown to be associated with high-risk neuroblastoma and unfavourable prognoses.^280–283^ Current research has not covered POC detection of these genes but this could be a potential avenue for research in the future.

##### 3.3.1.6 MicroRNAs

MiRNAs are made of 20–22 non-coding nucleotide molecules and are mainly found in the introns of protein-coding genes, and can control the expression of genes, such as *MYCN* and *TERT*.^284^ Studies have found there to be an upregulation of several miRNAs in aggressive forms of neuroblastoma.^285^ MiRNA proves to be resilient in harsh pHs and resistant to RNase activity, which allows it to be an effective detection tool.^286^ Several POC platforms have been developed for miRNA detection, including LFAs, electrochemical sensors, and portable qPCR systems. This shows the high feasibility of developing such a device for neuroblastoma in the near future.

#### 3.3.2 Proteomic biomarkers

##### 3.3.2.1 Neuro-specific enolase

Another biomarker that holds valuable prognostic information for neuroblastoma is neuron-specific enolase (NSE); a glycolytic protein found in neurons. NSE is elevated in patients with neuroblastoma, among other neuroendocrine tumours such as small-cell carcinomas and islet cell tumours.^287^ There is still a need to establish normal ranges for NSE, however it has been suggested that concentrations of this marker in serum remain between 16.95–26.71 ng mL^−1^ in healthy new-borns and 13.94–22.19 ng mL^−1^ in healthy infants.^288^ Such low concentrations make accurately measuring such biomarkers difficult, however given that NSE is not specific to neuroblastoma, it can be used as a prognostic predictor in patients with confirmed disease.^289^ NSE can be measured using assays such as enzyme-linked immunosorbent assay (ELISA). ELISA is a plate-based technique for detection and quantification of analyte measurement. While these devices are not POC and require trained professionals, they are very rapid and are widely used for the detection of disease biomarkers. NSE can be measured in biofluids including serum and pleural effusion fluid. Fan *et al.* developed a wireless POC testing system that involved testing NSE using microfluidic paper-based analytical devices, an electrochemical detector and smartphone.^290^ Comparing their results to ELISA, their device showed results within an acceptable range and has good selectivity towards NSE. Detection limits range from 1 to 500 ng mL^−1^ with a LOD of 10 pg mL^−1^, which would be suitable for NSE in neuroblastoma. Although this was developed for the detection of NSE in small cell lung cancer, this POC testing system proves to be promising for other tumours like neuroblastoma too. This suggests that POC assays, such as LFAs, are highly feasible for NSE detection. In terms of diagnosis of neuroblastoma, NSE cannot be used in isolation but could be used alongside the measurement of other biomarkers to confirm a diagnosis.

##### 3.3.2.2 Lactate dehydrogenase

Serum lactate dehydrogenase (LDH) is also used as a tumour biomarker for several cancers since it is indicative or tissue damage and has been shown to be elevated in *MYCN*-amplified neuroblastoma patients.^291^ High LDH values have been shown to correlate with poorer prognosis in both localised and metastatic disease states.^292^ In metastatic patients over the age of 18 months, LDH has recently been implicated as a predictor of poor event-free and overall survival rate.^293^ In new-borns, normal serum total LDH concentrations lie between 160 and 540 IU L^−1^.^294^ In children, serum LDH should be around 60–170 IU L^−1^.^294^ Studies have shown LDH to increase with increased tumour burden; thus, levels of the protein remain low in early stages of the disease.^15,49^ Separate studies have confirmed the strength of serum LDH in prognostication.^295^ Therefore, serum LDH measurement could be used as a diagnostic tool for high-risk neuroblastoma, although its ability to detect disease as an early stage is unknown. LDH can be measured using protein assays in samples of blood, cerebrospinal fluid or biopsies, as well as urine which provides a much less invasive alternative which is especially important for younger patients.^296^ However, assaying of biomarkers takes around 1–2 hours, and this does not qualify as POC; nonetheless, it is highly feasible that cheaper and more rapid devices such as LFAs could be developed for its detection.

#### 3.3.3 Chromogranin A

Chromogranin A is a known marker for neuroendocrine tumours due to its expression in the neuroendocrine system. In 1995, an ELISA to detect chromogranin A was developed specifically for diagnosis of neuroblastoma and a similar malignancy (phaeochromocytoma).^297^ It was found that 27% of neuroblastoma patients had normal levels of chromogranin A. Previous studies by Hsiao *et al.*^298^ suggested that chromogranin A was 91% sensitive for diagnosis of neuroblastoma; therefore, this ELISA test was not as sensitive as expected. The study concluded that the test added little benefit to current methods used in the diagnosis of these diseases at the time and that the measurement of plasma and urinary catecholamines had higher sensitivity overall. This analyte is co-released with catecholamines from storage vesicles;^298^ thus, it is not specific to high-risk neuroblastoma and therefore not a useful biomarker for implementation as a screening tool.

##### 3.3.3.1 Plasma-derived exosome proteins

Plasma-derived exosome proteins (exo-pro) show different properties depending on if the neuroblastoma is high or low risk.^299^ These differences can be seen in the proteins involved in cell migration, proliferation and metastasis. Exo-pro of neuroblastoma carry vital information about the primary origins of the neuroblastoma and has potential to be both a diagnostic and prognostic biomarker. Lateral flow immunoassays (LFIA) have been developed to test for exo-pros. Oliveira-Rodriguez *et al.*^300^ successfully developed an LFIA to analyse exo-pros from patient plasma. Each test can be done within 15 minutes. This was tested on metastatic melanoma cells but can be adapted to other cancers such as neuroblastoma.

##### 3.3.3.2 Vitronectin

Vitronectin is a glycoprotein important for cell migration. It is found in the plasma and extracellular matrix.^301^ Studies show higher levels of vitronectin are associated with poor neuroblastoma prognosis. The range of vitronectin in neuroblastoma patients range from 52.4 to 870 μg mL^−1^, with a suggested cut-off of 361 μg mL^−1^ as this is when patients over the age of 18 months had worse prognosis. As a new biomarker, more research is needed for the development of a POC device testing for vitronectin.

##### 3.3.3.3 Butyrylcholinesterase

Butyrylcholinesterase (BChE) is an enzyme that is altered in could neuroblastoma patients.^302^ When comparing BChE activity in *MYCN* amplified patients, this is around 40% lower than compared to non-MCYN amplified cases. BChE be a good biomarker to track treatments as its levels have been shown to normalise during treatment. In the context of burns and systemic inflammation, a POC testing device has been utilised in hospitals to measure BChE levels using blood samples.^303,304^ Thus, it is likely that such a device could be validated for neuroblastoma.

##### 3.3.3.4 Neuropeptide Y

Neuropeptide Y (NPY) is a sympathetic neurotransmitter. NPY is expressed in neuroblastoma, with elevated levels of NPY being associated with unfavourable outcomes of neuroblastoma.^305^ A novel sweat-detection based POC device has been developed for NPY detection in chronic anxiety orders, but the applicability of this to neuroblastoma needs to be assessed.^306^

##### 3.3.3.5 HLA-g

HLA-G is overexpressed in several tumours and is advantageous for tumour cell survival and development.^307^ ELISA tests have been developed for the detection of HLA-G^308^ which is overexpressed in neuroblastoma patients. In neuroblastoma, its levels also correlate with relapse, and thus a tool to rapidly measure this marker at POC could be used regularly by patients in remission, for earlier identification of relapse and improvement in outcomes for these patients. The use of such a device for neuroblastoma diagnosis, however, has not explored but could be a potential avenue of research for the future.

#### 3.3.4 Metabolic and biochemical biomarkers

##### 3.3.4.1 Polyamines

Polyamines are small molecules that regulate cell growth and differentiation. Thus, the polyamine pathway is implicated in cancer growth and progression, and in neuroblastoma, these molecules support processes regulated by the MYC family.^309^ Therefore, elevated levels of polyamines in patients could be used as a poor prognostic marker in children. Polyamines can be measured directly in blood or urine through detection of their metabolites, namely spermine, spermidine and putrescine. These molecules can be assayed in blood, saliva and urine samples,^310^ thus development of POC devices for their quantitation should not be difficult nor invasive. In the context of colorectal cancer, Igarashi *et al.* developed a multisegment injection capillary electrophoresis triple quadrupole tandem mass spectrometry (MSI-CE-MS/MS).^311^ This was used to detect polyamines to help differentiate between colorectal cancer, benign polyps and healthy controls. They successfully screened 359 salivary samples within 6 hours. This method proved to be sensitive and selective and shows promising applications within the field of POC screening. In polyamine synthesis, ornithine decarboxylase 1 (ODC1) is a rate-limiting enzyme involved in the conversion of ornithine to putrescine. ODC1 is also one of the transcriptional targets of *MYCN*, and thus high expression of ODC1 is found in *MYCN*-amplified neuroblastoma, linking this marker to poor outcomes in children.^309^ Another interesting consideration is the recent development of difluoromethylornithine (DFMO) which has been developed for inhibition of ODC1.^312^ Thus, a tool for the detection of ODC1 could be used not only for diagnosis, but also for monitoring of treatment efficacy.

##### 3.3.4.2 Catecholamines

Urine catecholamines, namely dopamine, epinephrine, and norepinephrine, are important liquid biomarkers for neuroblastoma since they, and their metabolites such as VMA and HVA, are elevated in patients. While studies have suggested that elevated VMA and HVA are linked to later stage disease, previous implementation of devices to screen for this were unsuccessful.^23,98,313^ HVA and VMA concentrations in urine should remain below 16.8 and 13.2 mmol mol^−1^ creatinine in infants under 6 months of age, respectively.^314^ Several assay methods are currently available for their detection, although liquid chromatography couples with tandem mass spectrometry (LC-MS/MS) is the gold standard for measurement of catecholamines and their metabolites.^315^ Detection of these analytes is one of the first-line diagnostic tests performed when a patient presents with symptoms of neuroblastoma and could be used in the future as part of multiplexed POC devices for rapid screening and diagnosis. However, catecholamines are elevated in the majority of neuroblastoma patients, and their rise is not linked to poorer outcomes, thus limiting their use in risk stratification.

##### 3.3.4.3 Glycosylated ferritin

Neuroblastoma cells secrete abnormal forms of ferritin (glycosylated ferritin). This biomarker is a poor prognostic marker for the disease, as levels of glycosylated ferritin are elevated mainly in stage 3 and 4S disease states.^316^ Levels of glycosylated ferritin can be measured in the blood using assays such as ELISA which are commercially available, and thus integrating this onto POC platforms should be possible.

##### 3.3.4.4 Serum cholesterol

Total serum cholesterol (Tchol) in neuroblastoma patients was analysed through a retrospective study.^317^ It found that neuroblastoma prognosis was associated with TChol and that it was significantly increased in patients with neuroblastoma that had relapsed and patients who died from neuroblastoma. The study concluded that TChol was also a risk factor for neuroblastoma as well as a prognostic biomarker. Traditionally TChol is measured through blood tests, but POC systems have been developed, but again, need to be validated in the context of neuroblastoma screening.^318^ Even though Tchol is not specific to neuroblastoma, it could contribute to a POC risk stratification panel combined with other, more specific biomarkers. Handheld, POC Tchol meters are widely available and can be implemented easily into a multiplexed biosensor.

##### 3.3.4.5 Neutrophil to lymphocyte ratio

Neutrophil to lymphocyte ratio is (NLR) is a marker of systemic inflammation for solid cancers.^319^ It has been shown to have prognostic capabilities in other cancers, such as oesophageal and squamous cell carcinomas and could possibly predict overall survival in paediatric solid tumours. The information to work out the NLR is part of the initial work up for diagnosis, so would not require further invasive testing, so NLR could be a potential useful prognostic biomarker in the neuroblastoma space. NLR can be calculated from complete blood counts, which is already feasible in portable haematology analysers. So, integrating these already developed technologies into a rapid tool for neuroblastoma risk stratification is practical.

##### 3.3.4.6 HALP scores

Haemoglobin, albumin, lymphocyte, and platelet (HALP) scores have been used as a prognostic marker in other solid tumours.^320^ It is calculated by the product of haemoglobin (g/L), albumin (g/L), lymphocyte count (per L) and platelet count (per L). Qi *et al.*^320^ conducted a retrospective study which showed a low HALP score was associated with poor prognosis. An optimal HALP cut off score was suggested at 27.0. Although invasive, as the components of the HALP score would need to be obtained through blood tests, these tests would be required anyway for a NB diagnosis, so a HALP score would be convenient to calculate.

#### 3.3.5 Cell surface of structural biomarkers

##### 3.3.5.1 Ganglioside GD2

Ganglioside GD2 is a glycolipid that is expressed on the membrane of NB tumours. It is shed into circulation, with its most predominant circulating form being C18 lipoform. Research has shown that ganglioside GD2 is a specific and sensitive tumour biomarker that can distinguish between high and low risk forms of NB, showing promising qualities for a diagnostic and prognostic NB biomarker.^321^ Balis *et al.* found that GD2 concentrations were significantly higher in patients with MYCN amplified tumours. Other studies corroborate this and showed that C18 and C20 forms of GD2 were higher in metastatic disease.^322^ Plasma concentrations of C18 and C20 were found to decrease during treatment, but increased during relapse, showing potential of GD2 monitoring treatment and disease. Current tests to detect GD2 in plasma involve high-pressure liquid chromatography/tandem mass spectrometry (HPLC/MS/MS), which can be adapted for clinical use.^321^

##### 3.3.5.2 Trk family

Tyrosine kinase receptors (Trk) play an important role in the development and clinical behaviour of NB.^323^ The Trk family consists of 3 Trks: Trk A, B and C. High expression of Trk A is associated with a good prognosis, with tumours displaying favourable features and is seen in tumours that spontaneously regress.^323,324^ On the other hand, high Trk B expression is associated with features such as MYCN amplification and unfavourable features and prognosis. The role of Trk C is less well known, but current literature shows that it is associated with positive outcomes.^324^ Tanaka *et al.*^325^ developed a immunohistochemical method to examine Trk A in tumours in a clinical setting. They concluded that Trk A was a strong predictor of good prognosis. This study however was performed in 1995 and little research on Trks as a biomarker has been conducted in recent times.

### 3.4 Challenges associated with diagnosing neuroblastoma at POC

Despite the clear advantages of POC devices for diagnosis of diseases, many issues can be faced in their development. Firstly, it is unlikely that POC technologies will completely replace current diagnostic devices, owing to the fact that they could not aid in identification of tumour location and may be of limited use for staging.^326^ However, they can improve time taken to diagnosis and be used as a companion to current methods.^9^ Developing POC devices with sufficient sensitivity to match gold-standard technologies is one of the biggest concerns; this is especially true for diseases for which the analyte may be in very low concentrations.^17^ For many biomarkers considered in this review, they are present in small quantities in blood and urine. One major disadvantage of POC devices is that samples should not be processed before detecting the biomarker as this will increase detection time. In infants, it is difficult to collect large enough blood samples, and urine tests rely on 24 hour collection.^327^ Sample enrichment or pre-concentration could be important for the detection of analytes, but this would increase detection times which should be a major consideration during development.^328^ Such issues can be overcome through the use of POC enhancement techniques, for example the use of fluorescent labels in LFAs or ELISA tests.^329^ Furthermore, since assays such as LFAs often utilise antibodies as detection reagents, cross-reactivity is a major concern. This has greater implications for childhood illnesses such as neuroblastoma, as false positive or negative results may lead to stress and anxiety for the child and their family, or even unnecessary invasive measures to investigate the child.^330^ Thus, the use of aptamers or methods which do not involve antibodies, such as chip technologies, could be a potential future move in the neuroblastoma field. Another major challenge would be validation of the device once it has been developed. A clinical trial would need to be undertaken to test that the device can be used routinely as a diagnostic device, and to compare its abilities to current methods.^331^ This would require a large sample size; however, neuroblastoma is a rare disease and recruiting enough subjects would be extremely difficult. The ability to overcome these issues and develop rapid POC devices for neuroblastoma diagnosis will be essential to the future management of this disease. The use of any developed tools for mass screening is a major concern, as it can lead to unnecessary interventions in children without hereditary germline risks. Thus, future development of diagnostic tools for neuroblastoma should focus on the differential diagnosis of high-risk disease.

## 4. Conclusion

Neuroblastoma is a clinically and biologically heterogeneous childhood malignancy, presenting mainly in babies and infants.^18^ The disease is characterised by significant morbidity in high-risk patients, which is often difficult to diagnose, and early screening attempts have been unsuccessful.^13,332^ Current diagnostic techniques for neuroblastoma rely on several biochemical, imaging, and histochemical tests. Patients often present to the hospital due to symptoms of the disease, most commonly palpable abdominal masses.^333^ From here, blood and urine tests showing hormone imbalances coupled with abnormal findings on CT/X-ray are enough to strongly suggest the diagnosis of neuroblastoma, although positive biopsy results are often required to confirm this.^21^ Thus, diagnosing neuroblastoma is extremely invasive and time-consuming, requiring several visits and causing significant stress and long-term health effects in young children. Furthermore, the cost of diagnosing neuroblastoma patients, and subsequent treatment of high-risk patients whose disease has progressed due to late diagnosis, is high. Over the past two decades, the diagnostic landscape of neuroblastoma has evolved significantly. Technological advancements have led to the discovery of several biomarkers, including genetic, proteomic and metabolic.^334^ Importantly, these diagnostic tools are increasingly being designed to be portable, cost-effective and rapid. Urine-based sensors for catecholamines and blood-based detection of exosomal proteins prove this.^158^ Furthermore, in integration of AI and machine learning algorithms holds great potential, and will allow researchers to automate interpretations, delivering rapid results without the requirement for specific equipment or training.^156^ The future of neuroblastoma is likely to rest on technologies that enable risk stratification at the bedside.

POC diagnostic devices have gained interest over recent years as they enable rapid diagnosis of disease at the patient's location, thus enabling more rapid implementation of interventions, saving time and money associated with long waiting times. Some POC devices such as LFAs are particularly useful since they are very cheap and rapid, and do not require trained professionals.^40^ In the future, POC devices can be manufactured to measure high-risk disease biomarkers such as MYCN protein and polyamine metabolites in biological samples.^335^ For other more generic biomarkers, such as catecholamine metabolites, this may also be useful as patients can use them at home and they can be used not only as diagnostic tools, but also for patient monitoring. Given that levels of biomarkers such as polyamine metabolites and catecholamine metabolites are known to be elevated in the blood and urine, and that this is well-documented in literature, developing and training POC devices is a logical step forward. Moreover, novel POC techniques are being developed and will continue to do so. Multiplexing is an emerging field of research which enables simultaneous biomarker measurement from a single sample at POC.^336^ This will ensure that for childhood diseases such as neuroblastoma, the impacts of false positive or negative results are minimised given that multiple analytes will be investigated before a conclusion is reached about the child's health. Such a device could act as a first step in patient care or as a screening tool in babies, helping to eliminate late diagnosis of this devastating disease.

## Conflicts of interest

The authors declare no competing financial interest.

## Data Availability

All data used in this review are available in the cited literature. No new datasets were generated.
